# Preparation, characterization and optimization of sildenafil citrate loaded PLGA nanoparticles by statistical factorial design

**DOI:** 10.1186/2008-2231-21-68

**Published:** 2013-12-19

**Authors:** Elham Ghasemian, Alireza Vatanara, Abdolhossein Rouholamini Najafabadi, Mohammad Reza Rouini, Kambiz Gilani, Majid Darabi

**Affiliations:** 1 Pharmaceutics Department, Faculty of Pharmacy, Tehran University of Medical Sciences, Tehran, Iran

**Keywords:** Sildenafil citrate, Nanoparticle, Optimization, Box-Behnken, Double emulsion

## Abstract

**Background and the aim of the study:**

The objective of the present study was to formulate and optimize nanoparticles (NPs) of sildenafil-loaded poly (lactic-co-glycolic acid) (PLGA) by double emulsion solvent evaporation (DESE) method. The relationship between design factors and experimental data was evaluated using response surface methodology.

**Method:**

A Box-Behnken design was made considering the mass ratio of drug to polymer (D/P), the volumetric proportion of the water to oil phase (W/O) and the concentration of polyvinyl alcohol (PVA) as the independent agents. PLGA-NPs were successfully prepared and the size (nm), entrapment efficiency (EE), drug loading (DL) and cumulative release of drug from NPs post 1 and 8 hrs were assessed as the responses.

**Results:**

The NPs were prepared in a spherical shape and the sizes range of 240 to 316 nm. The polydispersity index of size was lower than 0.5 and the EE (%) and DL (%) varied between 14-62% and 2-6%, respectively. The optimized formulation with a desirability factor of 0.9 was selected and characterized. This formulation demonstrated the particle size of 270 nm, EE of 55%, DL of 3.9% and cumulative drug release of 79% after 12 hrs. *In vitro* release studies showed a burst release at the initial stage followed by a sustained release of sildenafil from NPs up to 12 hrs. The release kinetic of the optimized formulation was fitted to Higuchi model.

**Conclusions:**

Sildenafil citrate NPs with small particle size, lipophilic feature, high entrapment efficiency and good loading capacity is produced by this method. Characterization of optimum formulation, provided by an evaluation of experimental data, showed no significant difference between calculated and measured data.

## Background

Sildenafil is a selective inhibitor of phosphodiesterase enzyme type 5 (PDE-5) that effectively inactivates cyclic guanosine monophosphate (cGMP) and enhances the effect of nitric oxide [1]. This drug was primarily prescribed for angina pectoris and now is widely used for the treatment of erectile dysfunction [2]. Recently, PDE-5 inhibitors have been proposed to protect the endothelial function in human by selectively improving local blood flow [3]. Moreover, other complications like wound healing, diabetic gastropathy [4], Reynaud’s phenomenon, respiratory disorders with ventilation/perfusion mismatch, congestive cardiac failure, hypertension and stroke have been widely studied with the hope that PDE-5 inhibitors can serve as novel promising treatment in such conditions. In addition, the selective and potent vasodilatory and antiproliferative effects of sildenafil on pulmonary vascular smooth muscle cells emphasize the importance of this drug in control of pulmonary artery pressure [4-6]. When sildenafil citrate (SC) is given orally, its bioavailability is relatively low (approximately 40%) in healthy subjects [7] because of the first pass metabolism. In addition, it exhibits a very short physiological half-life (about 3–4 hrs). Therefore repeated doses are required to sustain drug plasma level [8] that causes various side effects such as headache, flushing, dyspepsia and epistaxis [9].

Specially designed dosage forms that sustain levels of drug in the therapeutic window or local delivery of this drug into the site of action can be thus helpful [8,10]. Some published patents have reported that application of nano sized SC powder in formulation, shows faster onset of action, higher bioavailability and absorption than conventional dosage form [11-13]. Thus, nanoparticles (NPs) might improve the efficacy; reduce side effects and dosage of therapeutic agents [14]. Generally, nanocarrier systems provide advantages over conventional drug delivery systems such as protection of the entrapped drug from enzymatic destruction, sustained drug release, reduction of daily drug doses and side effects and cell targeting [15]. Consequently, biodegradable polymeric NPs have engrossed remarkable consideration as potential drug delivery devices in view of their applications in the controlled release of drugs [16]. poly(lactic-co-glycolic acid) (PLGA) is the most successfully used available biodegradable polymer due to its long clinical experience, desirable degradation characteristics and possibilities for sustained drug delivery [17]. This polymer is widely used in the production of NPs [18]. PLGA consists two endogenous monomers that are easily metabolized via the Krebs cycle and therefore, negligible systemic toxicity is associated with the use of PLGA for drug delivery [19]. Also, study of *in vitro* and *in vivo* cytotoxicity of PLGA nanoparticles highlighted the safety of biodegradable PLGA nanoparticles [20,21]. Selection of a particular method for preparation of NPs is usually determined by the solubility properties of the drug [22]. Double Emulsion Solvent Evaporation (DESE) technique is an effective method for encapsulation of hydrophilic compounds [23] and thus we selected this method to fabricate NPs, because of the polar properties of SC [24]. There are several variables in the DESE process that can affect the properties of the product. Response surface methodology (RSM) is a statistical method employed for the modeling and analysis of problems in which a response of concern is influenced by several variables and the goal is to optimize this response [25]. Application of such optimizing technique may be an efficient and economical method to gain the essential information and thus to understand the relationship between controllable independent variables and dependent variables or responses in terms of performance and quality [26].

## Methods

### Materials

PLGA, Resomer® RG503H, was acquired from Boehringer Ingelheim (Germany). Polyvinyl alcohol (PVA) (87–90% hydrolysis degree and molecular mass 30,000-70,000 g/mol) was purchased from Sigma Chemical Co. (USA). SC was purchased from Selleck Chemicals (USA). The organic solvents were supplied by Duksan (Korea).

### Preparation of SC-loaded Nanoparticles

At the first, the drug was dissolved in warm distilled water (3 mg/ml) and emulsified in methylene chloride containing different amounts of PLGA. The emulsification was carried out using a probe sonicator set (Hielscher, Germany) at 80% of the energy output for 3 min. Then, the primary emulsion was added to 20 ml of double distilled water containing PVA and homogenized for 3 min in 20,000 rpm (IKA, Germany). Methylene chloride was eliminated by evaporation under reduced pressure using a rotary evaporator (Buchi, Switzerland). NPs were recovered by ultracentrifugation (Beckman Instruments, USA) at 100,000 g for 60 min at 25°C.

### Experimental design

The effects of formulation variables on the NPs characteristics and optimization procedure were examined by employing a Box-Behnken design. The design and statistical analysis were performed by Design-Expert® V8 (DX8) Software for design of experiments (DOE). Experimental factors and factor levels were determined in preliminary studies. Studied responses which evaluated in this investigation, were the mass ratio of drug to PLGA (X_1_), the volumetric ratio of water to solvent in primary emulsion (X_2_) and the concentration of PVA (X_3_) that classified to low, medium, and high values for the chosen variables as are described in Table 1. The evaluated studied responses were size (nm), entrapment efficiency (EE), drug loading (DL) and drug release in 1 hr and 8 hrs. The Box-Behnken design and observational data are shown in Table 2.

**Table 1:** Factors and factor levels studied in a Box–Behnken experimental design

			**Levels**	
**Factors**		**Low (-1)**	**Medium (0)**	**High (1)**
**X**_**1**_	D/P	0.05	0.13	0.2
**X**_**2**_	W/O	0.25	0.38	0.5
**X**_**3**_	%PVA	0.1	0.55	1

**Table 2:** Run parameters and responses for three-level three-factorial Box–Behnken experimental design

**Runs order**	**D/P**	**W/O**	**PVA (%)**	**Size (nm)**	**EE (%)**	**DL (%)**	**Drug release in 1 hr**	**Drug release in 8 hrs**
**1**	1	0	-1	291	35	6.3	70	72
**2**	0	0	0	241	43	5.3	62	68
**3**	-1	0	1	316	51	3.2	30	39
**4**	1	0	1	259	28	5.1	79	79
**5**	0	-1	-1	259	26	3.1	75	80
**6**	0	0	0	279	47	5.8	49	50
**7**	0	-1	1	260	16	2	80	92
**8**	0	1	-1	304	32	4	51	59
**9**	0	1	1	311	32	4.1	36	43
**10**	-1	1	0	301	49	2.4	54	62
**11**	1	-1	0	246	14	2.5	92	93
**12**	1	1	0	261	26	4.8	79	81
**13**	0	0	0	268	45	5.5	77	86
**14**	-1	0	-1	303	62	3	29	40
**15**	0	0	0	260	45	5.6	49	65
**16**	-1	-1	0	278	41	2	61	75
**17**	0	0	0	240	46	5.6	50	64

The quadratic non-linear model generated by design is in this form:

$$\begin{array}{l} {Y=A}_{0}{+A}_{1}X_{1}{+A}_{2}X_{2}{+A}_{3}X_{3}{+A}_{4}X_{1}X_{2}{+A}_{5}X_{2}X_{3}\\ \;{+A}_{6}X_{1}X_{3}{+A}_{7}X_{1}{}^{2}{+A}_{8}X_{2}{}^{2}{+A}_{9}X_{3}{}^{2}+E \end{array}$$

In which Y is the measured response associated with each factor level combination; A_0_ is an intercept; A_1_-A_9_ are the regression coefficients; X_1_, X_2_ and X_3_ are the studied factors; X_1_^2^, X_2_^2^, X_3_^2^ are quadratic effects, X_1_X_2_ + X_2_X_3_ + X_1_X_3_ are interaction between variables and E is the error term [27].

### Physicochemical characteristics of NPs

#### Particle size

Mean hydrodynamic size (called z-average) and polydispersity index of the NPs were measured by photon correlation spectroscopy (Malvern, UK) at 25C. All the samples were diluted with double distilled water to create a suitable obscuration before analysis.

#### Determination of entrapped SC

The supernatant part of the centrifuged NP sample was carefully removed and examined to determine the amount of non-encapsulated drug. The precipitant was lyophilized, weighted, and then dissolved in a mixture of 3:2 of chloroform (a common solvent for PLGA) and water (a solvent for SC) by sonicating for one hour. Then, the undissolved fraction was removed by centrifugation. After that, a sample was taken from the aqueous phase to determine the amount of encapsulated SC. The drug incorporation efficiency was defined by the following formulas:

$$\mathrm{DL}\left(\%\right)=\frac{\text{Mass}\;\text{of}\;\text{sildenafil}\;\text{in}\;\text{NPs}}{\text{The}\;\text{mass}\;\text{of}\;\text{NPs}\;\text{recovered}}\;\times \;100$$

$$\text{EE}\left(\%\right)=\frac{\text{Mass}\;\text{of}\;\text{encapsulated}\;\text{sildenafil}}{\text{Mass}\;\text{of}\;\text{the}\;\text{total}\;\text{sildenafil}\;}\;\times \;100$$

A reverse phase chromatography method was used for evaluation of SC using isocratic HPLC system (Waters, USA) and NucleoDur (5 μm, 25 cm) C_18_ column. The mobile phase consisted of acetonitrile and water (35:65, pH 4.0) at a flow rate of 1 ml/min with UV detection at 291 nm. The retention time was 5.6 min.

#### Differential scanning calorimetry (DSC)

A differential scanning calorimeter (Mettler Toledo, Switzerland) was used to evaluate the thermal behavior of all materials used in the NP formulations. The equipment was calibrated using indium. The samples (8 mg) were heated ranging 5–280°C at a scanning rate of 10°C/min in aluminum pans under nitrogen gas.

#### Scanning electron microscopy (SEM)

The surface morphology of NPs was assessed by a scanning electron microscope (Mira Tescan, Czech Republic). The Nanoparticles were spread on a stub and dried at 25°C and then spattered with gold using a sputter coater (BAL-TEC, Switzerland).

#### In vitro drug release studies

To predict the optimal formulation of NPs, the drug release of each formulation was studied in phosphate buffer (PBS) at pH 7.4 as dissolution medium. Briefly, 10 mg of each lyophilized NP formulation was dispersed in a screw-capped glass vial (50 ml) containing 40 ml of medium by shaking at 200 rpm and 37 ± 0.5°C in shaker incubator (LABOTEC, Germany). At predetermined time intervals (0, 0.5, 1, 2, 4, 8, 10, 12, 24 hrs) 1 ml of the dispersion was taken away and replaced with 1 ml of fresh PBS. The sample was centrifuged (Eppendorf, Germany) at 14,000 g for 30 min, and the supernatant was analyzed. All of the experiments were done in triplicate.

The release kinetics from optimal NPs was fitted on zero order, first order, Higuchi model, Korsmeyer–Peppas model and Hixson–Crowell model [28].

## Results

NPs were successfully prepared by DESE method. The effects of formulation variables on the NP properties were evaluated and finally optimal NPs were proposed by design expert software. The characteristics of this formulation were compared to predicted values. In addition, release profile and release kinetics from optimal NPs were studied.

### Particle size

SC loaded NPs sizes varied between 240 to 316 nm. Formulations displayed polydispersity index (PDI) of <0.5 which showed the narrow NP size distribution.

**Figure 1:**
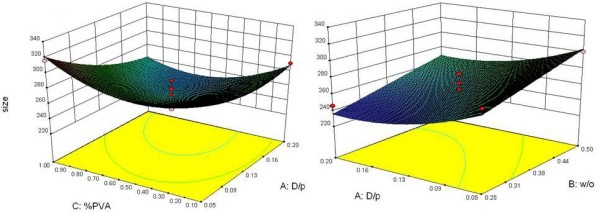
Surface plot showing the effect of different variables on particle size of SC nanoparticles.

Analysis of data from ANOVA test exhibited that D/P and W/O ratios had significant effects on particle size (p < 0.05). Briefly, decrease in the D/P and increasing the W/O ratios resulted in the production of larger particles (Figure 1).

In size response, the reduced model showed better adjusted correlation coefficients than the primary model (0.72 > 0.62) with an F value of 8 (p < 0.05), which clearly indicated that the particle size and some variables were related. The reduced model for predicting size is presented in equation 1.

(1) $$\begin{array}{c} \text{Particle}\;\text{size}=+258.68–17.63*X_{1}+16.75*X_{2}–1.38*X_{3}\\ \;+11.46*X_{1}{}^{2}+23.46*X_{3}{}^{2}–11.25*X_{1}*X_{3}\; \end{array}$$

The minimum particle size of 240 nm was achieved by operating the experiment at the midpoint of each independent variable. Analysis of independent factors showed that D/P and W/O ratios had effective impacts on particle size. There was no significant interaction between the studied factors.

### Determination of entrapped SC

The EE of NPs in different formulations is represented in Table 2. Data analysis of this response proved the significant effect of all independent variables (p < 0.05) and an interaction between W/O ratio and the amount of PVA (p < 0.05). The quadratic model of Entrapment Efficiency followed equation 2.

(2) $$\begin{array}{l} \mathrm{EE}=+45.20‒12.50*X_{1}+5.25*X_{2}‒3.50*X_{3}\\ \;+2.40*X_{1}{}^{2}‒15.10*X_{2}{}^{2}‒3.60*X_{3}{}^{2}\\ \;+1.00*X_{1}*X_{2}+1.00*X_{1}*X_{3}\\ \;+2.50*X_{2}*X_{3}\; \end{array}$$

**Figure 2:**
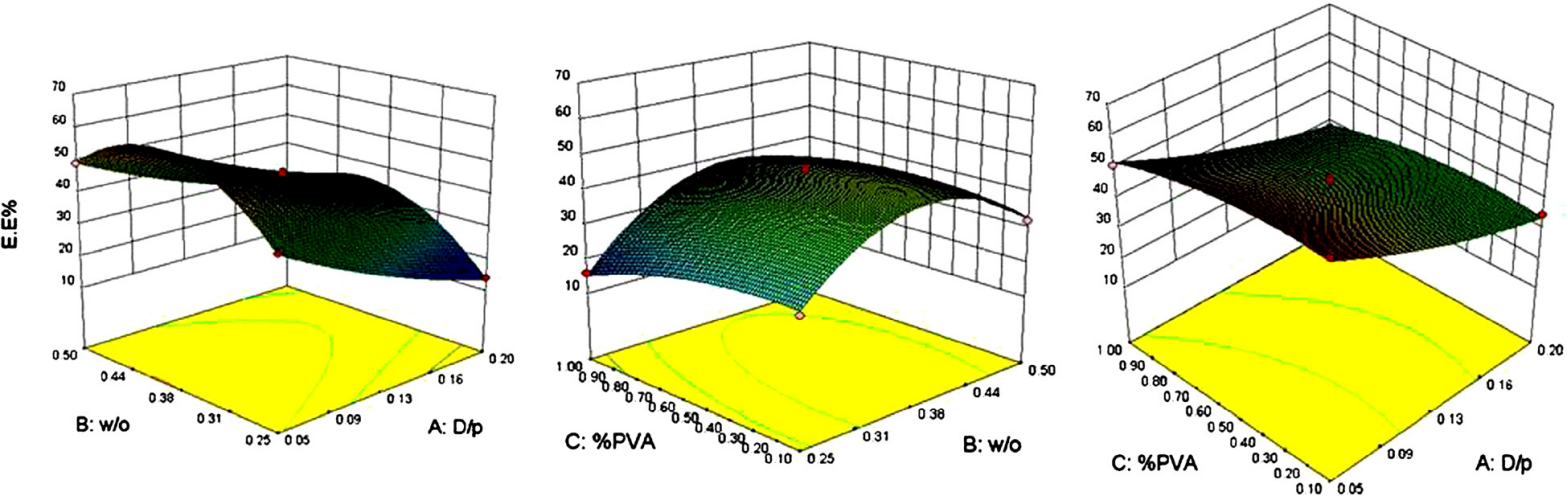
Three dimensional surface plots showing the effect of different variables on the %Entrapment Efficiency (EE) of SC nanoparticles.

Surface plots indicated that higher EE occurred in formulation with a W/O ratio about 0.38 and D/P ratios between 0.05-0.09. Also, PVA concentration was less effective than two other variables (Figure 2). The predictive model for DL is given in equation 3:

(3) $$\begin{array}{l} \mathrm{DL}=+5.56+1.01*X_{1}+0.71*X_{2}‒0.25*X_{3}-0.77*X_{1}{}^{2}\\ \;-1.87*X_{2}{}^{2}–0.39*X_{3}{}^{2}+0.48*X_{1}*X_{2}‒0.35*X_{1}*X_{3}\\ \;+0.30*X_{2}*X_{3}\; \end{array}$$

**Figure 3:**
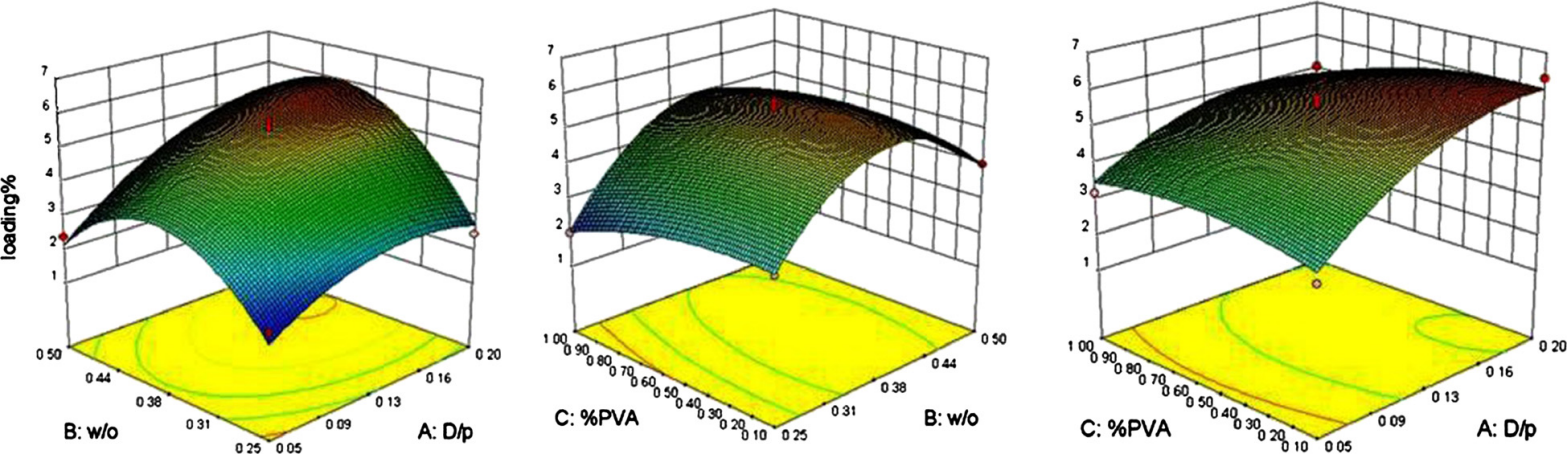
Three dimensional surface plots showing the effect of different variables on the %drug loading of SC nanoparticles.

DL in NPs ranged between 2% and 6.3% (Figure 3); where, analysis of data by ANOVA showed that the D/P Ratio with an F value of 71.36 (p < 0.05) had the most important impact on DL and W/O ratios had a significant effect on this response (p < 0.05). Comparison of different formulations and surface plots revealed that formulations with the highest DL had the maximum D/P ratio of 0.2 and a W/O ratio of approximately 0.38.

### *In vitro* drug release studies

To develop a formulation with acceptable release profile, drug release of each formulation was studied. Data of release over the first hour of experiments were considered as a marker of burst effect and the amount of drug releasing in 8 hrs showed retardation efficiency over the time. Analysis of the data demonstrated that W/O and D/P ratios had significant influence on release profiles.

For predicting the release in 1 and 8 hrs, the linear model fitted as an effective one with data (p < 0.05) (equations 4–5).

(4) $$\begin{array}{c} \%\mathrm{Release}\;1\;\mathrm{hr}=+60.18+18.25*X_{1}\\ \;-11.00*X_{2}+0.000*X_{3} \end{array}$$

(5) $$\begin{array}{c} \%\mathrm{Release}\;8\;\mathrm{hrs}=+67.53+13.63*X_{1}\\ \;-11.87*X_{2}+0.25*X_{3}\; \end{array}$$

**Figure 4:**
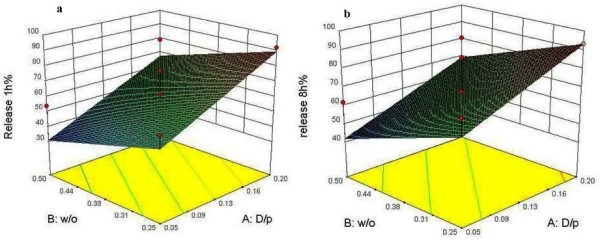
Three dimensional surface plots showing the effect of different variables on the release of drug from SC nanoparticles during 1 hr and 8 hrs.

Data analysis showed that the D/P ratios with F values of 17.08 and 9.10 (p < 0.05) were the most important factors on the release of SC from NPs during 1 and 8 hrs, respectively (Figure 4). When the D/P ratio was minimized and W/O ratio was about 0.38, the formulation had the least burst release. On the other hand, this formulation could provide sustained release profile over the time.

### Optimization

After confirming the polynomial equations relating the response and independent factors, in consequence of acceptable size of all formulations, the optimization model was constructed by combining the DL, EE and drug release in 1 hr responses. Optimization was performed by using a desirability function to obtain the levels of X_1_, X_2_ and X_3_, which maximized EE, while minimizing drug release in 1 hr and targeting DL at 4%. Coefficients with p-value < 0.05 had a significant effect on the prediction efficacy of the model for the measured responses. Simultaneously, the formulation with W/O about 0.40, D/P of 0.06 and PVA about 0.50 conformed higher desirability. This formulation prepared and evaluated. Predicted and actual amounts of responses are compared and shown in Table 3. As seen in Table 3, excluding release in 1 h, the amount of responses for optimized formulation have lower than 10% difference with the predicted amount of Box-behnken design.

**Table 3:** Comparison of actual and predicted properties of optimized NPs

	**Size (nm)**	**EE (%)**	**DL (%)**	**Release in 1 h (%)**	**Release in 8 hrs (%)**
**Predicted amount**	270	58.9	4	41	90.8
**Actual amount**	250	55	3.9	30	85
**Error (%)**	7.4	6.6	2.5	26	6.4

### Differential scanning calorimetry (DSC)

**Figure 5:**
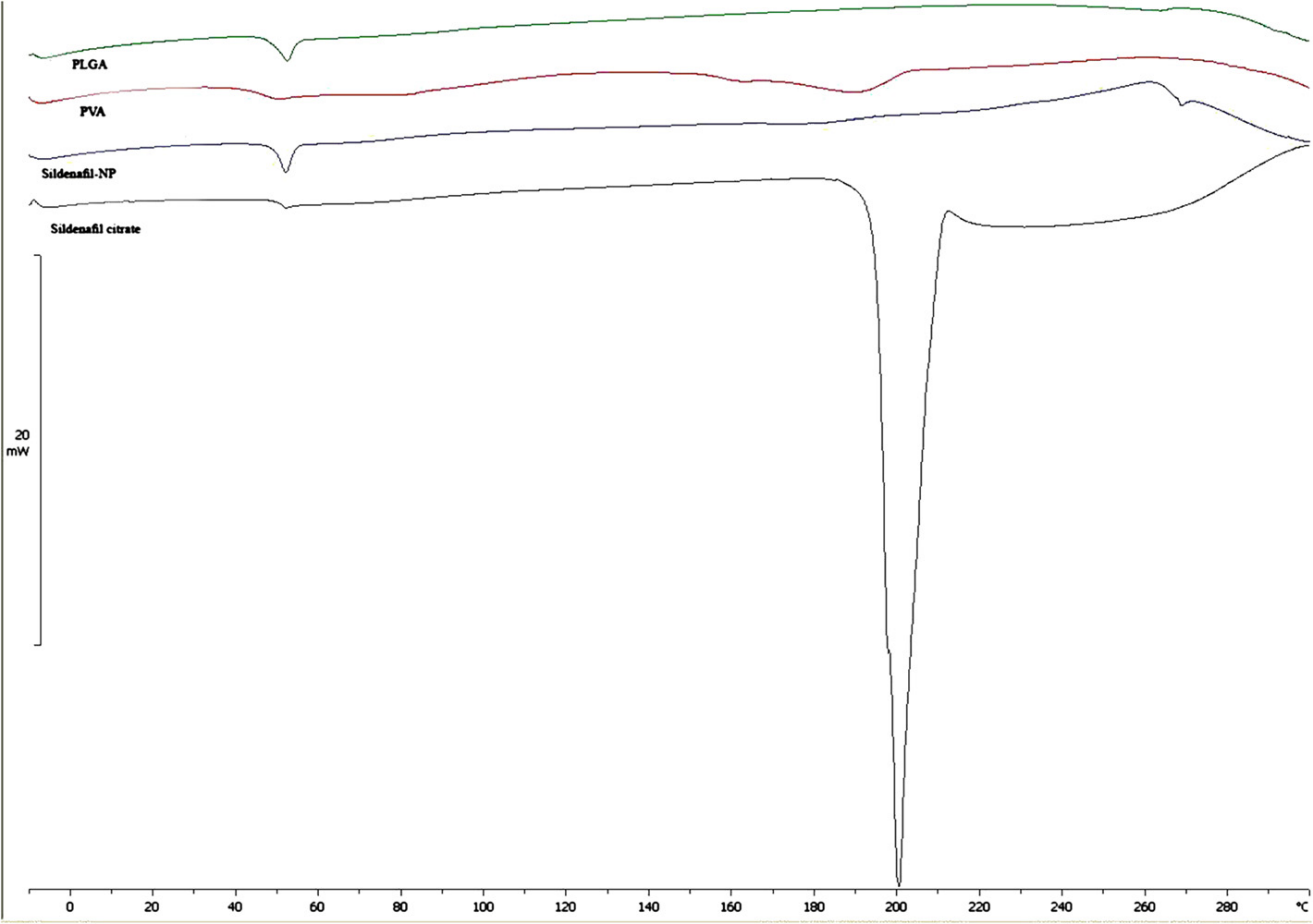
DSC thermogram of PLGA, PVA, Sildenafil citrate and Sildenafil-nanoparticles.

Thermal analysis is a supportive tool for determining the dispersion of the drug in polymeric materials. DSC thermograms of the pure drug, PVA, PLGA and SC-NPs are represented in Figure 5. The pure drug showed high endothermic peak indication of its melting peak at ∼ 200°C which was absent in NPs.

### Scanning electron microscopy (SEM)

**Figure 6:**
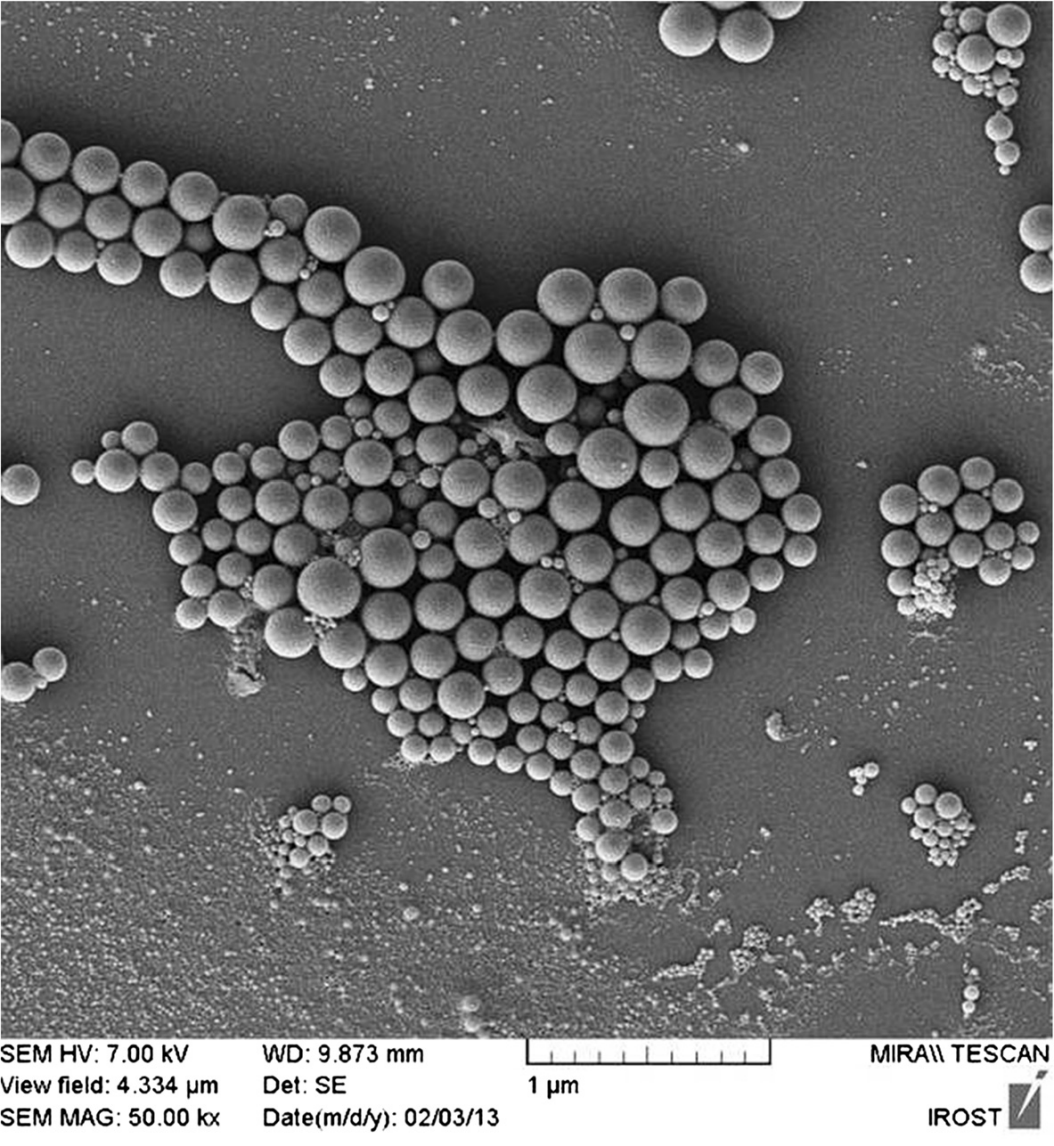
SEM micrograph showing the morphology of optimized PLGA-Sildenafil nanoparticles.

SEM micrographs showed that uniform PLGA NPs were successfully prepared by using the DESE method. As shown in Figure 6, the PLGA nanoparticles were in spherical shapes and a smooth surface.

### Release kinetics of optimal nanoparticles

**Figure 7:**
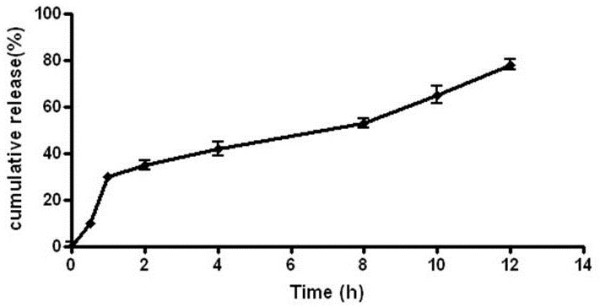
Profile of drug release from optimized SC nanoparticles.

Profile of release from optimal NPs is presented in Figure 7. *In vitro* drug release profiles of SLD from optimal PLGA NPs showed that the cumulative percentage of drug release was about 79% of drug content of the formulation in 12 hrs. The results support a burst release in the first one hour that followed by a sustained release over 12 hrs.

Release kinetics from the optimum formulation of NPs was compared to different kinetic models which showed that the best model fitted with data is the Higuchian equation (R^2^: 0.95). This model explains the release of drug from an insoluble matrix time-dependently based on Fickian diffusion [29]. The release constant was computed from the slope of the suitable plots, and the regression coefficient was determined (Table 4). The plots and regression coefficient proved that after Higuchian model, the best linearity followed by first-order kinetic (R^2^: 0.91). The first order kinetics model can be described the drug dissolution of water-soluble drugs in porous matrices [30].

**Table 4:** Results of model fitting for optimized Sildenafil NPs

**Models**	**Slope**	**R**^**2**^	**Intercept**
**Zero order**	0.002	0.772	0
**First order**	-0.106	0.925	4.496
**Higuchi**	21.44	0.950	0
**Korsmayer-Peppas**	0.51	0.860	3.044
**Hixon-Crowell**	0.187	0.656	2.264

## Discussion

Although the most applied method for encapsulation of hydrophilic drugs is DESE method, the low EE is usually a major problem. Experimental design methodology is an economic approach for extracting the maximum useful information from data. Applying this technique reduces the costs of experiments by saving time, materials and energy. Of course, optimization of NPs formulation is a complex procedure, which involves considering various parameters and their interactions. Due to increasing use of sildenafil in the treatment of pulmonary diseases and new indications proposed for this drug, preparation of optimum loaded NPs that release drug over the time can be potentially beneficial in the treatment of different pathological conditions.

Particle size and size distribution are important physicochemical properties that determine both uptake and biological fate of the particulate systems [31]. In the results, important effects of D/P and W/O ratios on size of NPs were confirmed. Production of NPs with higher polymer concentrations resulted in the formation of larger particles. In this manner, changing the diffusion rate of organic solvent through the interface could be proposed as a fundamental mechanism. In fact, increasing the amount of polymer or decreasing the volume of organic phase can potentially hinder the diffusion of solvent molecules through the polymeric chains [32]. Hence, formation of larger particles can be on account of two main factors: (a) the number of polymer chains per volume unit of solvent and (b) the viscosity of the solution [33]. As a result of applying a greater number of the polymeric chains per volume unit of solvent, diffusion of solvent into the aqueous phase becomes hard and causes the formation of aggregated and larger NPs, which is in agreement with previous reports [22,34]. On the other hand, it is more difficult for the viscous polymer solution to be broken up into smaller droplets during the formation of a second emulsion [35]. In this model, particle size reached to the minimum point when D/P and W/O ratios were about 0.13 and 0.36. Data analysis defined no significant effect of various PVA concentrations and this factor showed the minor influence on the particle size in this study. Of course, the presence of PVA as a surfactant is necessary to form stabilized NPs.

As mentioned in DESE method, the low EE of small and hydrophilic drug molecules into the polymer is an important challenge [36]. The water soluble nature of SC may be the cause of lower EE in higher D/P or W/O ratios. The higher EE values gained by the higher polymer contents can be explicated by the better coverage of drug molecules within the polymeric matrix [37]. In addition the initial amount of dissolved drug in the inner phase showed a great influence on EE. As the difference of drug concentrations between internal and external aqueous phase increases, the drug diffuses faster to the bulk aqueous phase during particle formation. In other words, higher DL values resulted in lower encapsulation efficiencies because of rapid partitioning of the drug between phases. So it can be claimed that DL and EE are strongly related responses [22,38]. In the formulations that the only changed parameter was W/O ratio, it seems that this factor had a dual effect. Although decreasing this ratio resulted in more efficiently covered aqueous droplets during first emulsion preparation, this larger amount of organic phase required much more time to evaporate [38]. So the drug molecules had greater opportunity to escape from the inner to outer phase in formulations with higher W/O ratios (>midpoint). While the aqueous volume was kept constant, the enhanced viscosity of the polymer solution led to the formation of larger polymer/solvent droplets. Consequently, slower solidification of larger particles allowed more drug diffusion to the external phase, which again resulted in the lower entrapment of the drug into the NPs [39]. Moreover, employing higher concentrations of stabilizer in the external aqueous phase induced higher EE values.

The endothermic peak of SC disappeared in the thermogram of optimized drug loaded NPs, which indicated absence of crystalline drug in the NPs. So, it can be assumed that encapsulated drug was in an amorphous state or a molecular dispersion throughout the polymer matrix after fabrication of NPs [40].

SEM micrographs showed smooth surface and spherical shape of SC-NPs which can be explained by stability of primary emulsion that the polymer had adequate time to form the condense matrix around the drug molecules before particle formation [41].

All formulations were subjects of *in vitro* release studies. The release profiles exhibited an initial burst release during the first hour, followed by a sustained release pattern over 12 hrs. Surface response plots showed the influence of D/P and W/O ratios on drug release from NPs. The initial burst release was related to the degree of DL, where the minor burst effects were observed in lower DL values. It appears that at higher loading levels, more drug molecules might adsorb onto the surface of NPs, which contribute to the greater initial release. Actually during the first hour of release study, the large concentration gradient of the drug serves as the driving force for the diffusion. But during the next hours not only this gradient decreases but also it takes more time for SC molecules to diffuse via a path constructed from a series of interconnected pores and channels within the polymeric matrix. It is supposed that in formulations with lower polymer concentrations, the internal water droplets have a greater tendency to coalescence and thus more likely to make larger pores and less tortuous network. However, when the higher polymer concentrations are applied, a tighter structure is formed as a result of faster droplet coagulation during second emulsion formation and subsequent polymeric chain entanglement. In another research published recently on the formation of sildenafil-NPs, the sildenafil was incorporated as its water insoluble base into the PLGA-NPs by means of solvent evaporation method, whereas the most of the entrapped drug released during the first 90 min [42]. Fortunately, in the present study, the release profile of sildenafil citrate as a hydrophilic salt with better bioavailability [1], improved up to 12 hrs.

## Conclusion

Comparing the actual and predicted responses indicated that surface response methodology is suitable to make optimization of SC NPs to produce a biphasic release pattern. These NPs can be utilized in the form of tablets or processed in the presence of inhalable sugars to form a dry powder for inhalation purposes. Further *in vivo* studies of SC NPs are recommended to determine whether oral, topical, transdermal and respiratory efficacies are created.

Although, providing SC in the form PLGA nanoparticles brings some advantages such as improvement in reaching many organs, tissues, and cells, the increased entrance into cells might fundamentally rises the chance of toxic effects. It is emphasized that changed size and surface area of the present nano form of sildenafil makes it prone to interact with various cellular components in various tissues. Therefore, future studies to collect the relationships between structure-size-efficacy-toxicity of the present nano form of sildenafil with special regard to portal of entry and target organ is crucial [43]. Additionally it would be nice to prove the safety of the new nano form of sildenafil in an appropriate biological system specially if the expectation is to use the new form of sildenafil for longer duration of time rather than its present single-dose indication in erectile dysfunction [44].

## Competing interests

The authors declare that they have no competing interests.

## Authors’ contribution

All authors have contributed significantly to the research and preparation, design and final production of the manuscript and approve its submission.
